# Factors Associated with Occurrence of Atelectasis during Sedation for Imaging in Pediatric Patients: A Retrospective Single Center Cohort Study

**DOI:** 10.3390/jcm10163598

**Published:** 2021-08-16

**Authors:** Pyeong Hwa Kim, Yong-Seok Park, Hee-Mang Yoon, Ah Young Jung, Eun-Young Joo, In-Cheol Choi, Myung-Hee Song

**Affiliations:** 1 Department of Radiology and Research Institute of Radiology, Asan Medical Center, University of Ulsan College of Medicine, Seoul 05505, Korea; peace4701@hotmail.com (P.H.K.); espoirhm@gmail.com (H.-M.Y.); akosjay@gmail.com (A.Y.J.); 2 Department of Anesthesiology and Pain Medicine, Asan Medical Center, University of Ulsan College of Medicine, Seoul 05505, Korea; parkys@amc.seoul.kr (Y.-S.P.); naeng82@gmail.com (E.-Y.J.); icchoi@amc.seoul.kr (I.-C.C.)

**Keywords:** magnetic resonance imaging, atelectasis, pediatric sedation, dexmedetomidine, propofol

## Abstract

Sedation can induce atelectasis which may cause suboptimal image quality. This study aimed to identify factors associated with the occurrence of atelectasis during sedation for imaging in pediatric patients. Patients < 18 years who had undergone whole-body magnetic resonance imaging (MRI) under sedation with propofol or dexmedetomidine were included in this study. The development of atelectasis was visually and quantitatively assessed by coronal short tau inversion recovery images of the thoracic level. Multivariable logistic regression was performed to identify the independent factors associated with the development of atelectasis. Ninety-one patients were included in the analysis. In the multivariable analysis, administration of supplemental oxygen was the only factor significantly associated with the occurrence of atelectasis (adjusted odds ratio, 4.84; 95% confidence interval, 1.48–15.83; *p* = 0.009). Univariable analysis showed that the use of dexmedetomidine was associated with a lower incidence of atelectasis; however, this could not be verified in the multivariable analysis. Among the pediatric patients who had undergone imaging under sedation, additional oxygen supplementation was the only independent factor associated with atelectasis occurrence. A prospective clinical trial is required to identify the cause-effect relationship between oxygen administration and occurrence of atelectasis during sedation.

## 1. Introduction

Prolonged sedation is inevitably required when performing imaging studies in young children who are uncooperative and more vulnerable to immobilization and noise exposure. Sedation during imaging improves the quality of images and enhances workflow efficiency [1,2,3]. However, it requires additional medical resources and costs. Furthermore, sedative agents may cause side effects, such as respiratory depression [1,4,5]. Atelectasis can develop during sedation and cause dyspnea or fever, requiring additional hospitalization [5,6,7,8,9]. Additionally, atelectasis may obscure pulmonary parenchymal lesions and be misinterpreted as pneumonia, metastasis, or lymph node enlargement. Therefore, it may increase the need for unnecessary additional imaging studies.

In pediatric imaging, propofol is a commonly used to induce sedation as it is safe and effective sedation for spontaneously breathing patients and has a fast onset and recovery [10,11,12]. In previous studies, the incidence of atelectasis in patients undergoing general anesthesia or sedation is reported as 20–94% depending on certain conditions. Additionally, the size of the atelectasis is reported as 2–4% of the total lung volume [6,9,13,14,15]. One study has reported that up to 82% of children develop atelectasis while undergoing magnetic resonance imaging (MRI) under light propofol-induced anesthesia with spontaneous breathing [6]. Dexmedetomidine is a highly selective alpha-2 agonist that induces natural sleep-like sedation with less respiratory depression; therefore, it has emerged as an alternative to conventional sedatives [12,16]. However, limited studies have evaluated the relationship between the use of dexmedetomidine and development of atelectasis in pediatric patients.

This study aimed to determine whether the type of sedative and administration of oxygen are associated with occurrence of atelectasis in pediatric patients undergoing whole-body MRI under sedation.

## 2. Materials and Methods

This study was approved by the institutional review board (IRB) of Asan Medical Center (IRB No. 2018-1485). The requirement for written informed consent was waived owing to the minimal risk to the study patients in this retrospective study.

### 2.1. Study Population

We performed a computerized search of the databases at our institution to identify patients who had undergone whole-body MRI under sedation between November 2017 and February 2018. During this period, two sedatives (propofol and dexmedetomidine) were used at the discretion of the anesthesiologist. Patients were included if they (a) were younger than 18 years, (b) underwent whole-body MRI under sedation with either propofol or dexmedetomidine, (c) had an American Society of Anesthesiologist Physical Status Classification of I or II, and (d) had available medical records including their medical history. Patients were excluded if they (a) were sedated with other sedatives or both propofol and dexmedetomidine, (b) had abnormalities in the thorax that interfered with the evaluation of the presence of atelectasis, or (c) underwent whole-body MRI not following our institution’s routine protocol.

### 2.2. Sedation Protocol

The process of sedation followed the routine protocol of the pediatric sedation clinic in our institution. Pre-sedation evaluation was performed during an anesthesiologist counseling session after a physical examination, which included the measurement of vital signs such as blood pressure, heart rate, body temperature (tympanic membrane temperature), and arterial oxygen saturation (using a pulse oximeter; SpO_2_). Volatile induction and maintenance of anesthesia with sevoflurane was performed for establishing intravenous access if needed.

For sedation, dexmedetomidine or propofol was chosen at the discretion of the anesthesiologist. Patients undergoing sedation with propofol received a bolus of 1 mg/kg propofol repeatedly until they became unconscious, followed by a continuous infusion of 100–200 mcg/kg/min of propofol to maintain sedation. Adjuvant agents including midazolam and/or ketamine were administered as required at the discretion of the anesthesiologist. Patients undergoing sedation with dexmedetomidine were infused a loading dose of 1.0–2.0 mcg/kg for 10 min, followed by a continuous infusion rate of 1.0–2.0 mcg/kg/h. For all patients, the target sedation level was modified Ramsey sedation scale level 5.

Before sedation, heart rate, blood pressure, SpO_2_, and partial pressure of end-tidal expiratory CO_2_ were monitored. After the patient became unconscious, their breathing was determined through direct observation and end-tidal CO_2_ monitoring by an anesthesiologist. If the patient’s breathing was not adequate, an oral airway was applied and chin lift was performed. MRI scanning was started after confirming the adequacy of the patient’s breathing. Oxygen was supplied via a nasal prong (2–4 L/min) or simple mask (6 L/min) as appropriate if the patient was hypoxic (SpO_2_ < 95%) despite spontaneous respiration being maintained. The sedation process was performed either directly by the attending staff in charge of the pediatric sedation clinic or by an anesthesiologist in residency or fellowship course under the supervision of the staff.

### 2.3. Image Acquisition

Whole-body MRI was performed using a 3T MR system (Ingenia, Philips Medical Systems, Best, The Netherlands) with a dedicated multichannel multi-element surface coil. Images were obtained using three to six subsequent table positions to cover the head to the toes, depending on the body size. All whole-body MRI procedures included coronal and sagittal short tau inversion recovery (STIR) imaging. When contrast enhancement was indicated, coronal non-enhanced T1-weighted fast spin echo images and post-contrast scans with coronal three-dimensional fat-suppressed T1-weighted gradient echo images were obtained. All scans were taken in spontaneously breathing patients.

### 2.4. Evaluation of Atelectasis

The development of atelectasis was visually assessed using initial and final coronal STIR images of the thoracic level obtained by a pediatric radiologist (HMY, 5 years of clinical experiences in pediatric body imaging), who was blinded to the medical records, including the anesthesia report. Atelectasis was graded according to the following five-point Likert scale: (1) Grade 1, no atelectasis; (2) Grade 2, linear atelectasis along the bronchovascular bundles; (3) Grade 3, crescent-like subpleural atelectasis; (4) Grade 4, segmental atelectasis; and (5) Grade 5, lobar atelectasis [6]. Additionally, atelectasis was quantitatively assessed by the pediatric radiologist (HMY). The margin of atelectasis was drawn on each image slice and volumetric calculations were automatically performed using the summation-of-area method of the picture archiving and communication system. The total lung volume was measured by drawing the margin of both lungs. Next, the atelectasis volume per the total lung volume (%) was calculated. During visual and quantitative analyses of atelectasis, the radiologist was blinded to the sedative used. The overall image quality for motion artifacts was assessed according to a five-point scale as follows: 1, unreadable; 2, extreme artifact; 3, moderate artifact; 4, mild artifact; and 5, no artifact.

The time intervals between induction of sedation and time to acquisition of first images (coronal STIR of thoracic level) and between first images and final image (coronal STIR of thoracic level) were also calculated.

### 2.5. Statistical Analysis

Continuous data are expressed as means ± standard deviations (SD) or medians with interquartile ranges. We compared parameters including the requirement of additional oxygen supplementation and rate of atelectasis development using the Chi-squared test. Multiple pairwise comparisons were conducted with Bonferroni correction. To identify independent factors associated with the development of atelectasis, univariable and multivariable logistic regression analyses were performed. Variables with *p*-values < 0.10 in univariable analysis were entered into the multivariable analysis. R software version 3.1.2 (R Foundation for Statistical Computing, Vienna, Austria) and MedCalc software (version 16.8, MedCalc, Mariakerke, Belgium) were used for statistical analyses. A *p*-value of <0.05 was considered statistically significant.

## 3. Results

### 3.1. Patient Characteristics

**Figure 1:**
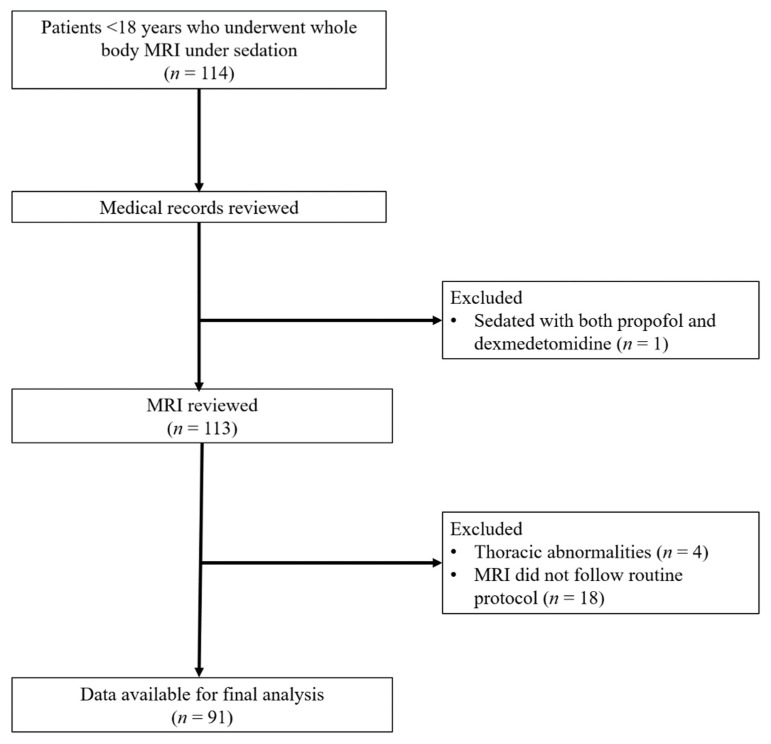
Study flowchart.

Among the 114 potentially eligible patients, one patient was sedated with both propofol and dexmedetomidine, four patients had thoracic abnormalities, and 18 patients underwent whole-body MRI that did not follow the routine protocol; therefore, they were excluded. Finally, 91 patients (mean age, 64 ± 41 months) were included (Figure 1). Baseline patient characteristics are listed in Table 1. Fifty-seven and 34 patients were sedated with propofol and dexmedetomidine, respectively. There was a significant difference in underlying disease incidence between propofol and dexmedetomidine group (*p* < 0.001): The propofol group had a higher proportion of neuroblastoma and leukemia/lymphoma, and the dexmedetomidine group had a higher proportion of neurofibromatosis Type I and Langerhans cell histiocytosis. More patients sedated with dexmedetomidine required adjuvant midazolam and/or ketamine than those sedated with propofol (44.1% [5/34] vs. 15.3% [9/59]; *p* = 0.003) (Table 1). The mean duration of the whole procedure was 56.5 ± 13.1 min (range, 20–87 min). According to the medical records, all patients recovered within one hour after cessation of sedation, and there were no sedation-related complications.

### 3.2. Supplemental Oxygen Administration and Rate of Atelectasis Occurrence

**Figure 2:**
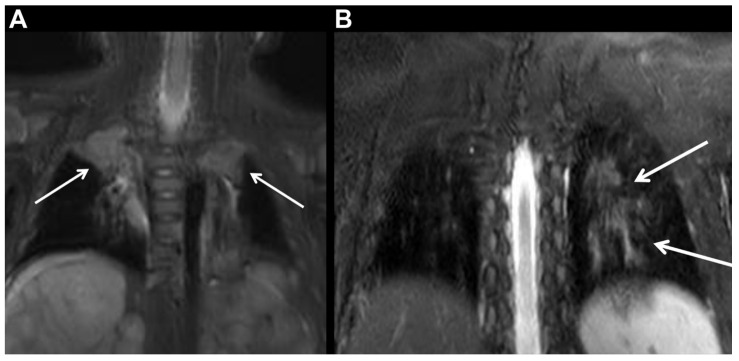
(**A**) A five-month-old male patient diagnosed with neuroblastoma and multiple hepatic metastases was sedated with intravenous propofol for whole-body magnetic resonance imaging. The initial image (coronal short tau inversion recovery image of thoracic level) shows segmental atelectasis (Grade 4; arrow) in both upper lobes. The estimated volume of atelectasis per the total lung volume was 13.6%. (**B**) A six-year-old male patient diagnosed with neurofibromatosis Type I was sedated with intravenous dexmedetomidine. The initial image (coronal short tau inversion recovery image of thoracic level) shows linear atelectasis along bronchovascular bundles (Grade 2; arrow) in the left lower lobe. The estimated volume of atelectasis per the total lung volume was 1.08%.

Thirty-seven of 57 patients (64.9%) sedated with propofol needed additional oxygen supplementation during sedation, while only one of 34 patients (2.9%) sedated with dexmedetomidine required additional oxygen supplementation during sedation (*p* < 0.001). Further, atelectasis occurred more frequently in patients sedated with propofol than in those sedated with dexmedetomidine (45.6% [26/57] vs. 17.6% [6/34]; *p* = 0.007) (Table 2). Representative cases are presented in Figure 2.

Grade 2 atelectasis was the most common pattern observed in patients sedated with either propofol or dexmedetomidine (Table 3 and Table 4). Patients sedated with propofol who needed additional oxygen supplementation more frequently developed a higher grade of atelectasis than those who did not need additional oxygen supplementation (*p* = 0.015 on initial images and *p* = 0.013 on final images; both Bonferroni-adjusted *p* < 0.017) or those sedated with dexmedetomidine (*p* = 0.016 on initial images and *p* < 0.001 on final images; both Bonferroni-adjusted *p* < 0.017). There was no significant difference between the propofol without additional oxygen supplementation and dexmedetomidine groups. Three patients who had Grade 4 atelectasis on initial images had no clinically relevant adverse event after whole-body MRI examination. The median volume of atelectasis per the total lung volume (%) was not significantly different between the three groups. During imaging acquisition under sedation, the volume of atelectasis tended to increase in patients sedated with propofol, while it tended to decrease in patients sedated with dexmedetomidine (Table S1 in Supplementary Materials).

### 3.3. Factors Associated with Development of Atelectasis

Univariable analysis revealed that sedative type, requirement of additional oxygen supplementation, and induction time had a *p*-value of <0.1; therefore, they were entered into the multivariable analysis. Among the three variables, the requirement of additional oxygen supplementation was the only significant factor affecting the occurrence of atelectasis (adjusted odds ratio, 4.838; 95% confidence interval, 1.478–15.831; *p* = 0.009) (Table 5).

## 4. Discussion

Our multivariable regression analysis revealed that additional oxygen supplementation was the only independent factor associated with the occurrence of atelectasis in pediatric patients who had undergone MRI under sedation. Use of dexmedetomidine had a lower odds ratio for the occurrence of atelectasis in the univariable analysis; however, this was not verified in the multivariable analysis.

Atelectasis caused by anesthesia is associated with several mechanisms including absorption atelectasis, tissue compression, and decreased surfactant function [17,18,19]. Absorption atelectasis is caused by the uptake of gas from the alveoli into the blood because of airway occlusion or decrease in the ventilation/perfusion ratio. The functional residual capacity (FRC) is reduced in the supine position and can be further worsened by anesthesia exposure, resulting in airway occlusion, and trapped gas is absorbed causing atelectasis [17]. A reduction in FRC during anesthesia occurs regardless of whether the patient receives mechanical ventilation or is breathing spontaneously and whether inhaled or intravenous anesthesia is used [17,20]. Compression atelectasis is a phenomenon in which the lungs are collapsed by compression against lung tissue, causing gas in the alveoli to be squeezed out. According to some studies, compression atelectasis during general anesthesia is caused by changes in the position and movement of the chest wall and the diaphragm owing to the loss of respiratory muscle tone [18,21,22]. We hypothesize that absorption atelectasis is the main cause of atelectasis in the patients in our study because they were breathing spontaneously with relatively intact muscle tone. Further, we found that oxygen administration was independently associated with the development of atelectasis, which was an important finding in our study.

Oxygen therapy is used in many clinical situations. However, supplemental oxygen administration is associated with detrimental side effects including airway inflammation, worse neurologic outcomes, and increased mortality in specific clinical situations [23,24,25]. Oxygen supplementation is also associated with the occurrence of atelectasis. In a study of patients undergoing general anesthesia, breathing 100% oxygen was associated with significantly larger atelectasis areas than breathing room air. Additionally, hyperoxygenation performed by changing the inhaled oxygen concentration from 40% to 100% significantly increased the extent of atelectasis [26]. In another study, atelectasis developed more frequently in patients receiving 100% oxygen than in those receiving 80% or 60% oxygen during preoxygenation for general anesthesia [27]. However, in this study, patients were under sedation rather than general anesthesia, and the fraction of inspired oxygen was relatively low using a nasal cannula or a simple mask. Moreover, the oxygen may have been supplied after the occurrence of atelectasis, which could act as a confounding factor. Therefore, it is difficult to conclude that the association between oxygen supply and atelectasis in this study indicates a causal relationship that oxygen supply itself was the sole cause of atelectasis.

Propofol is a commonly used sedative agent in pediatric patients; it is rapid and effective for various diagnostic tests and therapeutic procedures [11,28,29,30]. In a randomized prospective study involving pediatric patients undergoing MRI, propofol had a significantly shorter time to onset of sedation, recovery time, and discharge time than dexmedetomidine [12]. However, the disadvantages of propofol include pain from injection, which is the most common adverse effect [31]; hypotension due to a reduction in systemic vascular resistance and reduced cardiac output [32]; and propofol infusion syndrome, which is a rare but critical complication with high mortality [33]. Propofol reduces FRC, which increases the risk of hypoxia and atelectasis in children [34,35]. In addition, propofol induces respiratory depression and upper airway obstruction [11,36], leading to the high incidence of supplemental oxygen administration in patients who received propofol in our study. By contrast, dexmedetomidine causes little respiratory depression [37]. In a study of pediatric patients undergoing MRI, children who were sedated with dexmedetomidine showed less respiratory depression and no desaturation compared with those who were sedated with propofol [12]. Furthermore, dexmedetomidine does not cause respiratory acidosis or desaturation even at supramaximal plasma concentrations [38]. However, few studies have assessed the relationship between dexmedetomidine administration and changes in FRC or development of atelectasis.

In our study, adjuvant sedatives were more frequently used in the dexmedetomidine group than in the propofol group. This may be because the induction time of dexmedetomidine was longer, the dose used in this study was insufficient, or it was difficult to reach target sedation level. Benzodiazepines reduce respiratory drive in a dose-dependent pattern and mildly decrease FRC, while ketamine usually preserves respiratory drive and FRC [34]. However, the use of adjuvant sedatives was not associated with the occurrence of atelectasis in our study. The relationship between the use of adjuvant sedatives and the development of atelectasis may need to be studied separately.

There were no clinical symptoms or complications caused by atelectasis in our study. Atelectasis can be a direct or indirect cause of perioperative complications such as fever, pneumonia, bronchospasm, and respiratory failure [39]. While these complications usually occur in severe atelectasis, most cases in our study had linear atelectasis. This study included patients with American Society of Anesthesiologist Physical Status Classification of I or II; therefore, it may be possible that patients with poor general condition may develop severe atelectasis with symptoms or complications. Moreover, the presence of atelectasis interferes with the imaging of other pulmonary diseases, such as parenchymal lesions, reducing the diagnostic ability of imaging tests [40]. In the clinical practice of pediatric radiology, sedation-related atelectasis is seen occasionally and can be problematic in cases where unnecessary additional rescanning or follow-up imaging can be required. This could result in additional exposure to sedatives and/or radiation and increase in medical costs. Therefore, reducing sedation-related atelectasis is vital in pediatric chest imaging.

This study has several limitations: (1) As a retrospective study, unexpected biases cannot be excluded. (2) The choice of sedative agent was not systematically randomized; therefore, the effect of confounding factors is possible. Additionally, there was a significant difference in underlying diseases between the propofol dexmedetomidine group; therefore, a selection bias for the choice of sedatives cannot be excluded. Moreover, the anesthesiologist who decides on the type of sedative was not blinded to the diagnosis of patients because he/she takes the patients’ medical history during pre-examination consulting. Propensity matching, which could have minimized these biases and confounding factors, was unavailable due to the small study population. (3) A target level of sedation was set; however, no objective depth of sedation (e.g., bispectral index) was monitored. (4) Vital signs, including oxygen saturation were recorded manually without electronic medical recording system. This would have provided more detailed and precise in-formation on the oxygen requirement.

## 5. Conclusions

Among the pediatric patients who had undergone whole-body MRI under sedation, the only significant independent factor associated with the occurrence of atelectasis was additional oxygen supplementation. A prospective randomized clinical trial designed to exclude selection bias and confounding factors is required to identify the cause-effect relationship between oxygen administration and atelectasis during sedation.

## Figures and Tables

**Table 1:** Baseline patient characteristics.

	Propofol	Dexmedetomidine	*p*-Value
No. of patients	57	34	
Age (months) *	66.9 ± 43.5	56.2 ± 33.6	0.219
Male:female	27:30	19:15	0.432
Height (cm) *	107.6 ± 23.6	103.2 ± 19.9	0.362
Weight (kg) *	20.5 ± 11.8	18.8 ± 8.3	0.818
Time interval between induction of sedation and initial image scanning (min) *	17.1 ± 5.7	23.0 ± 5.3	<0.001
Time interval between initial and final image scanning (min) *	37.8 ± 12.8	36.5 ± 8.6	0.568
Overall image quality score on initial images *	4.6 ± 0.6	4.4 ± 0.6	0.284
Overall image quality score on final images *	4.6 ± 0.6	4.34 ± 0.5	0.263
Use of O_2_	37 (64.9%)	1 (2.9%)	<0.001
Use of adjuvant agents	9 (15.3%)	15 (44.1%)	0.003
Underlying disease			<0.001
Neuroblastoma	17 (29.8%)	4 (11.8%)	
Neurofibromatosis Type I	12 (21.1%)	23 (67.6%)	
Leukemia/lymphoma	10 (17.5%)	0	
Langerhans cell histiocytosis	8 (14.0%)	7 (20.6%)	
Other ^†^	10 (17.5%)	0	

Note: data are presented as number of patients, unless otherwise specified. * Data are presented as mean ± standard deviation. ^†^ Other diseases included Ewing’s sarcoma (*n* = 2), ganglioneuroblastoma, infantile fibrosarcoma, peripheral neuroblastic tumor, Gaucher disease, Niemann–Pick disease, neurofibromatosis Type II, lymphangioma, and Rosai-Dorfman disease (each, *n* = 1).

**Table 2:** Proportion of supplemental oxygen administered and rate of atelectasis occurrence according to the sedative agent used.

	Supplemental Oxygen Administered	Atelectasis on Initial Images	Atelectasis on Final Images	Atelectasis on Either Initial or Final Images
Propofol	37/57 (66.7%)	21/57 (36.8%)	25/57 (43.9%)	26/57 (45.6%)
Dexmedetomidine	1/34 (2.9%)	6/34 (17.6%)	5/34 (14.7%)	6/34 (17.6%)
*p*-value	<0.001	0.053	0.004	0.007

**Table 3:** Rate of atelectasis occurrence according to the atelectasis grade on initial images.

Group		*n*	Atelectasis Grade						*p*-Value ^†^		
			1	2	3	4	5	Any Atelectasis	I	II	III
I	Propofol + O_2_ (+)	37	19 (51.4%)	14 (37.8%)	1 (2.7%)	3 (8.1%)	0	18 (48.6%)	NA	0.015	0.016
II	Propofol + O_2_ (−)	20	17 (85.0%)	3 (15.0%)	0	0	0	3 (15.0%)	0.015	NA	0.534
III	Dexmedetomidine	34	28 (82.4%)	4 (11.8%)	2 (5.9%) *	0	0	6 (17.6%)	0.016	0.534	NA

NA = not applicable. Atelectasis grade: 1 = no atelectasis, 2 = linear atelectasis along bronchovascular bundles, 3 = crescent-like subpleural atelectasis, 4 = Segmental atelectasis, 5 = lobar atelectasis; * one patient who required oxygen supply was included in this category. ^†^ In the Chi-square test, atelectasis grade was significantly different between the three groups (*p* = 0.021). Post hoc analysis showed that a higher grade of atelectasis occurred more frequently with Propofol + O_2_ (+) than with Propofol + O_2_ (−) (*p* = 0.015; Bonferroni-adjusted *p* < 0.017) and dexmedetomidine (*p* = 0.016; Bonferroni-adjusted *p* < 0.017).

**Table 4:** Rate of atelectasis occurrence according to the atelectasis grade on final images.

Group		*n*	Atelectasis Grade						*p*-Value ^†^		
			1	2	3	4	5	Any Atelectasis	I	II	III
I	Propofol + O_2_ (+)	37	17 (45.9%)	12 (32.4%)	6 (16.2%)	2 (5.4%)	0	20 (54.1%)	NA	0.013	<0.001
II	Propofol + O_2_ (−)	20	15 (75.0%)	5 (25.0%)	0	0	0	5 (25.0%)	0.013	NA	0.556
III	Dexmedetomidine	34	29 (85.3%)	4 (11.8%) *	1 (2.9%)	0	0	5 (14.7%)	<0.001	0.556	NA

NA = not applicable. Atelectasis grade: 1 = no atelectasis, 2 = linear atelectasis along bronchovascular bundles, 3 = crescent-like subpleural atelectasis, 4 = Segmental atelectasis, 5 = lobar atelectasis; * one patient who required supplemental oxygen was included in this category. ^†^ In the Chi-square test, atelectasis grade was significantly different between three groups (*p* = 0.011). Post-hoc analysis showed that a higher grade of atelectasis occurred more frequently with Propofol + O_2_ (+) than with Propofol + O_2_ (−) (*p* = 0.013; Bonferroni-adjusted *p* < 0.017) and dexmedetomidine (*p* < 0.001; Bonferroni-adjusted *p* < 0.017).

**Table 5:** Logistic regression analysis of factors associated with the occurrence of atelectasis.

Parameters	Univariable Analysis			Multivariable Analysis		
	Unadjusted OR	95% CI	*p*-Value	Adjusted OR	95% CI	*p*-Value
Age (months)	1.008	0.997–1.019	0.157			
Sex						
Female	Reference category			Reference category		
Male	0.797	0.334–1.888	0.606			
Sedative type						
Propofol	Reference category			Reference category		
Dexmedetomidine	0.255	0.085–0.677	0.009	0.828	0.204–3.350	0.791
Supplemental oxygen administration	5.912	2.304–15.173	<0.001	4.838	1.478–15.831	0.009
Induction time (min)	0.931	0.856–1.006	0.072	0.970	0.891–1.055	0.473
Scan time (min)	1.018	0.980–1.058	0.361			
Use of adjuvant agents	0.692	0.252–1.899	0.474			

OR = odds ratio; CI = confidence interval. Variables with *p* < 0.1 were entered into the multivariable analysis.

## Data Availability

The data presented in this study are available on request from the corresponding author. The data are not publicly available due to the policy on data utilization research.

## Supplementary Materials

The following are available online at https://www.mdpi.com/article/10.3390/jcm10163598/s1, Table S1: quantitative analysis of atelectasis volume; Table S2: logistic regression analysis of factors associated with the occurrence of atelectasis in a subgroup of patients not requiring supplemental oxygen administration; Table S3: logistic regression analysis of factors associated with the occurrence of atelectasis in a subgroup of patients sedated with propofol.
