# Supplementary material for: Factors Associated with Occurrence of Atelectasis during Sedation for Imaging in Pediatric Patients: A Retrospective Single Center Cohort Study
Source: J Clin Med. 2021 Aug 16;10(16):3598. doi: 10.3390/jcm10163598 (PMC8397091; doi:10.3390/jcm10163598)
Supplement: Supplementary file 1 [file jcm-10-03598-s001.zip › jcm-1287602-supplementary.pdf]

Table S1. Quantitative analysis of atelectasis volume

Group	Median atelectasis volume per total lung volume on initial images (%)	Median atelectasis volume per total lung volume on final images (%)	P-value*
Propofol + O_2 (+)	1.15 (0.2–2.0)	1.29 (0.8–4.1)	0.242
Propofol + O_2 (-)	0.94 (0–0.9)	1.17 (0.9–1.3)	0.401
Dexmedetomidine	1.06 (0.4–1.7)	0.63 (0.3–1.2)	0.310
P-value†	0.748	0.240	

Data are presented as medians with interquartile ranges.

* P-values of Wilcoxon signed rank test for comparison between initial and last images.

† P-values of Kruskal–Wallis test for comparison of the three groups.

Table S2. Logistic regression analysis of factors associated with occurrence of atelectasis in subgroup of patients not requiring supplemental oxygen administration (n = 53)

Parameters	Univariate			Multivariate		
	Odds Ratio	95% CI	P-value	Odds Ratio	95% CI	P-value
Age (per 1 month)	0.996	0.974–1.018	0.721			
Sex						
Female	1					
Male	1.048	0.265–4.149	0.947			
Drug						
Propofol	1			1		
Dexmedetomidine	0.536	0.134–2.149	0.379	0.501	0.094–2.665	0.418
Induction time	0.977	0.856–1.114	0.729	1.011	0.866–1.181	0.886
Scan time	1.010	0.945–1.079	0.767			
Use of adjuvant agents	1.381	0.335–5.695	0.655			

Table S3. Logistic regression analysis of factors associated with occurrence of atelectasis in subgroup of patients sedated with propofol (n = 57)

**Parameters**	**Univariate**			**Multivariate**		
	**Odds Ratio**	**95% CI**	**P-value**	**Odds Ratio**	**95% CI**	**P-value**
Age (per 1 month)	1.006	0.994–1.019	0.307			
Sex						
Female	1					
Male	0.914	0.322–2.598	0.866			
Supplemental oxygen administration	3.937	1.182–13.113	0.026	3.957	1.185–13.218	0.025
Induction time	0.969	0.88–1.066	0.517	0.968	0.88–1.065	0.508
Scan time	1.015	0.974–1.059	0.472			

Use of adjuvant agents	0.945	0.226–3.959	0.939
